# Combination of Cyclosporine A and Levosimendan Induces Cardioprotection under Acute Hyperglycemia

**DOI:** 10.3390/ijms22094517

**Published:** 2021-04-26

**Authors:** Carolin Torregroza, Birce Yueksel, Raphael Ruske, Martin Stroethoff, Annika Raupach, André Heinen, Markus W. Hollmann, Ragnar Huhn, Katharina Feige

**Affiliations:** 1 Department of Anesthesiology, Medical Faculty and University Hospital Duesseldorf, Heinrich-Heine-University Duesseldorf, Moorenstr. 5, 40225 Duesseldorf, Germany; Carolin.Torregroza@med.uni-duesseldorf.de (C.T.); Birce.Yueksel@hhu.de (B.Y.); Raphael.Ruske@hhu.de (R.R.); Martin.Stroethoff@med.uni-duesseldorf.de (M.S.); Annika.Raupach@med.uni-duesseldorf.de (A.R.); KatharinaKristina.Feige@med.uni-duesseldorf.de (K.F.); 2 Institute of Cardiovascular Physiology, Medical Faculty and University Hospital Duesseldorf, Heinrich-Heine-University Duesseldorf, Universitaetsstr. 1, 40225 Duesseldorf, Germany; Andre.Heinen@med.uni-duesseldorf.de; 3 Department of Anesthesiology, Amsterdam University Medical Center (AUMC), Location AMC, Meiberdreef 9, 1105 AZ Amsterdam, The Netherlands; M.W.Hollmann@amsterdamumc.nl

**Keywords:** Levosimendan, hyperglycemia, myocardial infarction, cardioprotection

## Abstract

Prognosis of patients with myocardial infarction is detrimentally affected by comorbidities like diabetes mellitus. In the experimental setting, not only diabetes mellitus but also acute hyperglycemia is shown to hamper cardioprotective properties by multiple pharmacological agents. For Levosimendan-induced postconditioning, a strong infarct size reducing effect is demonstrated in healthy myocardium. However, acute hyperglycemia is suggested to block this protective effect. In the present study, we investigated whether (1) Levosimendan-induced postconditioning exerts a concentration-dependent effect under hyperglycemic conditions and (2) whether a combination with the mitochondrial permeability transition pore (mPTP) blocker cyclosporine A (CsA) restores the cardioprotective properties of Levosimendan under hyperglycemia. For this experimental investigation, hearts of male Wistar rats were randomized and mounted onto a Langendorff system, perfused with Krebs-Henseleit buffer with a constant pressure of 80 mmHg. All isolated hearts were subjected to 33 min of global ischemia and 60 min of reperfusion under hyperglycemic conditions. (1) Hearts were perfused with various concentrations of Levosimendan (Lev) (0.3–10 μM) for 10 min at the onset of reperfusion, in order to investigate a concentration–response relationship. In the second set of experiments (2), 0.3 μM Levosimendan was administered in combination with the mPTP blocker CsA, to elucidate the underlying mechanism of blocked cardioprotection under hyperglycemia. Infarct size was determined by tetrazolium chloride (TTC) staining. (1) Control (Con) hearts showed an infarct size of 52 ± 12%. None of the administered Levosimendan concentrations reduced the infarct size (Lev0.3: 49 ± 9%; Lev1: 57 ± 9%; Lev3: 47 ± 11%; Lev10: 50 ± 7%; all ns vs. Con). (2) Infarct size of Con and Lev0.3 hearts were 53 ± 4% and 56 ± 2%, respectively. CsA alone had no effect on infarct size (CsA: 50 ± 10%; ns vs. Con). The combination of Lev0.3 and CsA (Lev0.3 ± CsA) induced a significant infarct size reduction compared to Lev0.3 (Lev0.3+CsA: 35 ± 4%; *p* < 0.05 vs. Lev0.3). We demonstrated that (1) hyperglycemia blocks the infarct size reducing effects of Levosimendan-induced postconditioning and cannot be overcome by an increased concentration. (2) Furthermore, cardioprotection under hyperglycemia can be restored by combining Levosimendan and the mPTP blocker CsA.

## 1. Introduction

Myocardial infarction (MI) usually presents as an unforeseeable event that is still associated with high rates of mortality and morbidity [1,2]. Even after survival of MI, patients are faced with a substantial risk of subsequent cardiovascular events, including sudden cardiac arrest or heart failure [3]. Prognosis after suffering from MI widely varies among patients and is immensely affected by comorbidities like hypertension or diabetes [4,5]. Interestingly, hyperglycemia seems to be an independent outcome-related risk factor for MI in patients with or without diabetes [6,7,8].

Restoration of coronary blood circulation is essential for all patients suffering from MI [9]; however, the paradoxically occurring ischemia-reperfusion (I/R) injury hampers the benefits of reperfusion. I/R injury is characterized as additional cell damage and death caused by restored blood supply to an ischemic organ or tissue, like the myocardium [10]. Hence, cardioprotective interventions protecting the heart against harmful consequences of I/R injury are fundamental. Considering the fact that MI—as an acute event—is mostly unpredictable, cardioprotective approaches performed after the occurrence of ischemia and subsequent I/R injury gain increasing relevance. Zhao et al. [11] described the mechanism of ischemic postconditioning (PoC), where short cycles of ischemia and reperfusion after a prolonged ischemic period induced a significant myocardial protection. Fortunately, a less invasive practice—administration of pharmacological agents—can mimic ischemic PoC. Different substances were demonstrated to confer myocardial protection, e.g., sedatives [12], opioids [13], or the calcium (Ca^2+^) sensitizer—Levosimendan [14,15].

In a clinical setting, Levosimendan is indicated for the treatment of acute and chronic heart failure, significantly lowering mortality when administered preoperatively, with the benefit of having positive inotropic characteristics without affecting the diastolic function [16]. The molecular background of Levosimendan relates to its specific interaction with cardiac troponin C in myofilaments, resulting in an increased sensitivity for Ca^2+^ in cardiomyocytes [17]. Experimental studies suggest that Levosimendan-induced conditioning is mediated via activation of mitochondrial potassium (mK^+^) channels [15,18,19] and phosphatidylinositol 3-kinase (PI3K) [20], ultimately causing a decrease of infarct size and improved cardiac function after I/R injury [15,21].

However, various comorbidities abolish cardioprotection induced by ischemic and pharmacological postconditioning [22,23,24]. In particular, acute hyperglycemia—which is frequently observed in patients with cardiovascular diseases—blocks several known conditioning strategies [24,25,26,27,28]. In line with these findings, Matsumoto et al. demonstrated that hyperglycemia raised the threshold for postconditioning with Levosimendan in vivo. A ten times higher concentration of Levosimendan was needed to restore the cardioprotective effect [29]. Authors suggest an involvement of known cardioprotective targets, like mK^+^ channels or the mitochondrial permeability transition pore (mPTP), in the hyperglycemia-induced blockade. However, definite evidence is lacking to this point. The mitochondria are thought to be the end-effector of cardioprotection [30,31] and the mPTP is considered to be the most integral player [32,33]. Opening of the mPTP ultimately leads to matrix swelling and cell death caused by the release of proapoptotic factors [34]. Referring to the influence of hyperglycemia, Huhn et al. [22] demonstrated that applying the mPTP inhibitor cyclosporine A (CsA) fully restores Sevoflurane-induced postconditioning, under elevated glucose levels.

Therefore, in this study, we set out to determine whether (1) acute hyperglycemia influences concentration-dependent postconditioning with Levosimendan and (2) if the combination with CsA reverses the hyperglycemia-caused loss of cardioprotection by Levosimendan.

## 2. Results

### 2.1. Animal Characteristics

Regarding body weight, wet and dry weight, and level or time of maximal contracture, no differences were detected between groups in both parts of this study (Table 1). For part 2 of the study, one dropout had to be reported in the Con group. The experiment was excluded from the study as it did not meet the baseline hemodynamic criteria (low baseline heart rate). Referring to the onset of maximal ischemia contracture, no differences were detected between the experimental groups. These results illustrate that myocardial damage caused by global ischemia was comparable between all study groups, underlining that differences in infarct size resulted from the respective postconditioning stimulus and not by varying the myocardial ischemic damage.

### 2.2. Infarct Size

**Figure 1:**
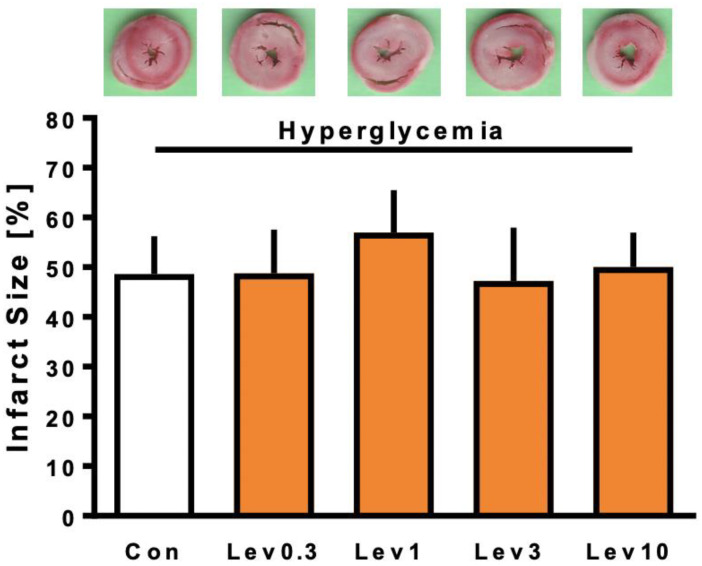
Infarct size measurement part 1. Histogram shows all infarct sizes of the study. Data are presented as means ± SD.

Infarct sizes of part 1 of the study are shown in Figure 1. Infarct size of control hearts was 52 ± 12%. Levosimendan (Lev) in a concentration of 0.3 µM did not reduce infarct size under hyperglycemia (Lev0.3: 49 ± 9%; ns vs. Con). Increasing Levosimendan concentration also did not induce infarct size reduction (Lev1: 57 ± 9%; Lev3: 47 ± 11%; Lev10: 50 ± 7%; all ns vs. Con).

**Figure 2:**
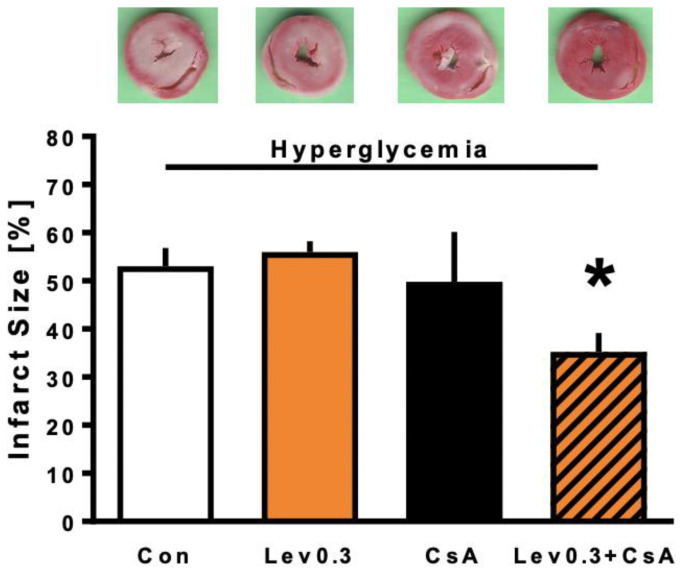
Infarct size measurement part 2. Histogram shows all infarct sizes of the study. Data are presented as means ± SD, * *p* < 0.05 vs. Lev0.3.

All infarct sizes investigated in part 2 are displayed in Figure 2. Control and Lev0.3 hearts showed infarct sizes of 53 ± 4% and 56 ± 2% of the left ventricle, respectively. Combining Lev0.3 with the mPTP blocker CsA (Lev0.3 ± CsA) significantly reduced the infarct size compared to Lev0.3 (Lev0.3+CsA: 35 ± 4%; *p* < 0.05 vs. Lev0.3). The mPTP blocker itself had no effect on infarct size (CsA: 50 ± 10%; ns vs. Con).

### 2.3. Cardiac Function

In part 1 of the study, no differences were observed between the groups at the different measurement time-points. Heart rate remained stable throughout the whole experimental protocol. A significant decrease during reperfusion compared to baseline was detected for both left ventricular, which developed pressure and coronary flow values within each group. All hemodynamic variables from part 1 can be found in Table 2.

Table 3 displays all hemodynamic variables from part 2 of the study. Again, no differences were detected between the different groups for any given time-point of measurement. Comparable to part 1 of the study, during reperfusion pressure and coronary flow that developed in the left ventricular decreased significantly, as compared to the baseline within each study group.

### 2.4. Glucose Levels

All glucose levels are displayed in Table 4. Compared to baseline, all groups showed a significant increase in glucose values (hyperglycemia). For part 1 and 2 of the study, there were no differences in glucose concentration between the individual groups.

## 3. Discussion

In the present study, we focused on the influence of hyperglycemia on postconditioning with Levosimendan and whether increased concentrations or combined inhibition of mPTP could overcome a potential loss of cardioprotection. Our results demonstrated that (1) acute hyperglycemia fully abrogated Levosimendan-induced postconditioning even under increased substance concentrations. However, (2) combining the mPTP blocker CsA and Levosimendan restored cardioprotection under acute hyperglycemia.

### 3.1. Influence of Hyperglycemia on Levosimendan-Induced Postconditioning

Acute hyperglycemia is not only considered an independent risk factor of cardiovascular diseases, like MI, but also significantly influences morbidity and mortality after occurrence of ischemia and reperfusion [4,6,8]. More importantly, elevated glucose levels crucially interfered with several ischemic and pharmacological conditioning strategies in experimental studies [24,35,36]. This might be one explanation for the remaining challenge of successfully translating cardioprotective approaches into the clinical setting [37,38]. Therefore, unraveling the influence of hyperglycemia on conditioning strategies is of considerable importance.

In a previous study, we demonstrated a concentration-related cardioprotective effect of postconditioning by Levosimendan [14]. We detected an on–off phenomenon, which was either ineffective or had maximal effect under normoglycemic conditions. A concentration of 0.3 μM Levosimendan as postconditioning stimulus induced cardioprotection, whereas an increase to 1 μM had no additional effect. Results from our own and other studies indicate that Levosimendan-induced postconditioning is mainly mediated via the reperfusion injury salvage kinase (RISK) pathway [18,20]. In more detail, mitochondrial adenosine triphosphate (ATP)-sensitive potassium (mK_ATP_) and large conductance calcium-sensitive potassium (mBK_Ca_) channels are suggested to be main downstream targets of cardioprotection by Levosimendan [14,15,19,20]. All of these targets are possibly blunted by elevated glucose levels [23,39,40].

Previous studies on hyperglycemia and ischemic or pharmacological conditioning indicated that the loss of cardioprotection can be reversed by increased stimuli. In an in vitro I/R animal study, one cycle of ischemic preconditioning did not reduce infarct size, while three cycles of the same stimulus did confer cardioprotection in diabetic myocardium [41]. The same holds true for pharmacological preconditioning with increased concentrations of Isoflurane [42].

Matsumoto et al. [29] previously demonstrated that hyperglycemia blocks postconditioning with Levosimendan in vivo, presumably due to a raised threshold for cardioprotection. This explanation was based on findings that increased concentration of Levosimendan did indeed induce infarct size reduction under elevated glucose levels. However, a ten times higher concentration was needed to achieve cardioprotection. In contrary to these results, in our present study even increased concentration did not induce cardioprotection under hyperglycemia in vitro. A possible explanation might be the different experimental settings. Matsumoto et al. employed an in vivo I/R animal model, while our experiments were performed in isolated hearts in vitro. We chose this setting to exclude possible systemic influences of other organs or hormones. Furthermore, our study involved hyperglycemia throughout the whole 60 min reperfusion phase. In contrast, Matsumoto et al. applied the glucose solution only during the first half of reperfusion, with a total of 120 min reperfusion phase. These differences in experimental protocol as well as possible systemic influences in vivo vs. in vitro might explain the contrary findings in our present study.

Nevertheless, increasing concentrations of Levosimendan to overcome blocked protection by hyperglycemia might not be advisable for patients. We showed that 0.3 µM was the lowest cardioprotective concentration of Levosimendan under normoglycemia in vitro. This converted to around 100 µg/L which was in line with the detected plasma concentrations (10–100 µg/L) under clinical dosage of Levosimendan [43,44,45]. However, it was already situated at the higher end of the therapeutic dose range. Considering adverse effects, applying a ten times higher concentration of Levosimendan under hyperglycemia does not seem practical in the clinical setting. Thus, increased dosage to restore blocked cardioprotection through hyperglycemia might not be feasible for Levosimendan-induced postconditioning.

### 3.2. Reversing the Loss of Levosimendan-Induced Cardioprotection under Hyperglycemia by Combined Treatment with CsA

From the literature, it is well-known that acute hyperglycemia attenuates cardioprotection; however, detailed explanation of possible underlying mechanisms is still lacking. Studies indicate that hyperglycemia leads to elevated ATP levels, which in turn hampers activation of the mK_ATP_ channels [39,46,47]. Next to ATP, excessive levels of reactive oxygen species (ROS) were detected under hyperglycemic conditions [48]. While ROS are essential in conferring cardioprotection, disproportional amounts ultimately cause opening of the mPTP and thus cell death [30,49]. Acute hyperglycemia not only has a negative impact on the mK_ATP_ channels but also blocks different parts of cardioprotective signaling cascades, for example, Akt phosphorylation, nitric oxide (NO), endothelial NO synthase (eNOS), or protein kinase G (PKG) [23,25,50,51]. Some of these respective targets are crucially involved in Levosimendan-induced cardioprotection. Especially, regulation of mitochondrial bioenergetics through mK^+^ channels and mPTP seems to play an integral role in conditioning strategies, under hyperglycemia [39]. Matsumoto et al. [29] demonstrated blocked postconditioning with Levosimendan under elevated glucose levels, while Milrinone was still effective. They presented involvement of different mK^+^ channels as a possible explanation for this discrepancy. Levosimendan is mediated via both mBK_Ca_ and mK_ATP_ channels [14,18]. Milrinone, however, is supposedly only dependent on mBK_Ca_ channels. Both substances ultimately target the mPTP and the protective properties could be fully abolished by administration of the mPTP activator atractyloside [29]. Taking together all these findings, it could be assumed that regulation of mitochondrial function, in particular mK_ATP_ channels, ROS levels and mPTP, play an integral role in lost cardioprotective properties of Levosimendan, under hyperglycemic conditions.

Multitarget strategies, meaning combination of substances or conditioning approaches, were the main research focus in the context of overcoming challenges in translating cardioprotection into clinical trials [52]. Next to the above-mentioned increased stimulus or concentration, combined conditioning strategies were shown to restore cardioprotection under hyperglycemia. Kehl et al. demonstrated that hyperglycemia blocks Isoflurane-preconditioning, but combination with the ROS scavenger N-acetylcystein restores the cardioprotective effects [50]. These findings further underline the importance of ROS in hyperglycemia. Interestingly and in line with our findings, cardioprotection with Sevoflurane is completely abrogated under acute hyperglycemia. However, inhibiting mPTP opening by administration of CsA, reversed the loss of protective effects [22]. Similar to our results, Huhn et al. [22] demonstrated that applying CsA individually did not restore cardioprotection under hyperglycemia. Even though inhibition of mPTP opening with CsA was shown to protect healthy myocardium [53,54], elevated glucose levels seemed to interfere with these properties. Further, they also demonstrated that an increased concentration of CsA did not overcome the hyperglycemia-induced loss of cardioprotection. These findings indicate that a pharmacological stimulus could possibly amplify the inhibition of mPTP with CsA and thus induce cardioprotection, even under hyperglycemia. Another possible explanation might be a lowered threshold for cardioprotection by combining stimuli triggering different pathways. Our results were consistent with findings from Huhn et al. on Sevoflurane and CsA [22]. In this study and to our knowledge for the first time, we demonstrated that while hyperglycemia blocks pharmacological conditioning with Levosimendan, this loss of cardioprotection could be restored with co-administration of CsA.

### 3.3. Limitations

While our results showed a significant infarct size reduction by combining Levosimendan and CsA under acute hyperglycemia, no hemodynamic improvement was detected during reperfusion, as compared to the other study groups. The concept of myocardial stunning after global ischemia might explain these rather contradictory findings. Myocardial stunning is defined as a temporary depression of function in the surviving myocardial tissue, which occurs especially after global ischemia. Even a prolonged reperfusion phase (up to 120 min) only displayed slight changes in hemodynamic data—insufficient for assessment of heart function—with no further impact on infarct size reduction [55]. Consistent with current literature, measurement of infarct size still represents the most sensitive marker to access cardioprotection in the isolated heart [56]. Based on these aspects, we chose the respective experimental protocol of our study.

Furthermore, we did not conduct experiments investigating the underlying mechanisms of restored cardioprotection by combining Levosimendan and CsA under hyperglycemia. Whether beneficial effects by simultaneous treatment were achieved by a lowered threshold of the same mitochondrial end-effector or parallel activation of different signaling pathways, remains an open question at this point. Investigating these exact underlying mechanisms were beyond the scope of our current study. Further research is needed to unravel whether mPTP is completely blocked by combining Levosimendan and CsA or if other pathways are involved.

Lastly, due to animal ethical reasons, we refrained from including normoglycemic control groups. We [14] and others [18] previously demonstrated that Levosimendan-induced postconditioning significantly reduced infarct size in healthy isolated hearts. Moreover, our own research group investigated the underlying mechanisms of postconditioning with Levosimendan by employing the exact same experimental setup. Stroethoff et al. demonstrated that Levosimendan-induced postconditioning reduced infarct size by about −50%, as compared to a normoglycemic control group [14]. Hence, these previous results could be referenced for normoglycemic groups, in the context of Levosimendan-induced postconditioning.

## 4. Materials and Methods

All experiments included in this study were conducted in accordance with the Guide for the Care and Use of Laboratory Animals published by the U.S. National Institute of Health (NIH publication No.85-23, revised 1996). Investigations were approved by the local Animal Care and Use Committee of the University of Duesseldorf (project number O27/12), and results were reported according to the ARRIVE guidelines.

### 4.1. Surgical Preparation

The surgical preparation was performed as described in detail previously [57]. Male wistar rats (2–3-month-old) were anesthetized with intraperitoneal injection of pentobarbital (80 mg/kg body weight, Narcoren, Merial, Germany) and decapitated. Hearts were resected via a thoracotomy, mounted onto a Langendorff-System (built in-house) and perfused with Krebs-Henseleit-Buffer (KHB) (Chemicals sourced from Sigma-Aldrich, Germany; KHB solution prepared in-house) enriched with a mixture of 95% O_2_ and 5% CO_2_. The KHB solution contained 118 mM NaCl, 4.7 mM KCl, 1.2 mM MgSO_4_, 1.17 mM KH_2_PO_4_, 24.9 mM NaHCO_3_, 2.52 mM CaCl_2_, 11 mM glucose, and 1 mM lactate, and was perfused under constant pressure (80 mmHg) and temperature (37 °C). For hemodynamic measurements, a fluid-filled balloon (manufactured in-house) was inserted into the left ventricle, setting left ventricular end-diastolic pressure to 4–6 mmHg. For all experiments, heart rate, left ventricular end-systolic pressure (LVESP), left ventricular end-diastolic pressure (LVEDP), and coronary flow (CF) were continuously measured and digitized by an analogue to digital converter (PowerLab/8SP, ADInstruments Pty Ltd., Castle Hill, Australia), at a sampling rate of 500 Hz. LVESP and LVEDP values allowed for calculation of left ventricular developed pressure (LVDP) (LVDP = LVESP − LVEDP). As a possible indicator for differences in myocardial injury, we analyzed the level and time-point of maximal contracture during ischemia for each experiment. To examine infarct size, after successful completion of the protocol, each heart was removed and cut into 8 transverse slices (2 mm each slice). Afterwards, a 0.75% triphenyltetrazoliumchloride (TTC) solution was applied to detect the infarcted area as compared to viable tissue. A blinded, experienced investigator analyzed infarct sizes using planimetry (SigmaScan Pro5 software), determined as the percentage of infarct area per total area of the left ventricle [58].

### 4.2. Experimental Protocol

**Figure 3:**
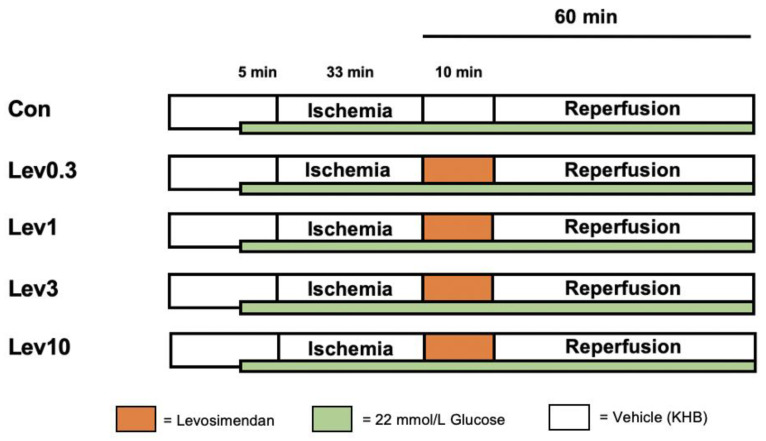
Experimental protocol. Con = Control; Lev = Levosimendan, KHB = Krebs-Henseleit-Buffer; Vehicle = Krebs-Henseleit Buffer (KHB). **Green bar:** Hearts were perfused with a total of 22 mmol/L glucose concentration by combining 11 mmol/L glucose solution with KHB (containing 11 mmol/L glucose). **Control (Con):** Hearts were perfused with Krebs-Henseleit-Buffer (KHB) as vehicle for 10 min. **Levosimendan 0.3 μM (Lev0.3):** Hearts were perfused with 0.3 μM Lev for 10 min. **Levosimendan 1 μM (Lev1):** Hearts were perfused with 1 μM Lev for 10 min. **Levosimendan 3 μM (Lev3):** Hearts were perfused with 3 μM Lev for 10 min. **Levosimendan 10 μM (Lev10):** Hearts were perfused with 10 μM Lev for 10 min.

**Figure 4:**
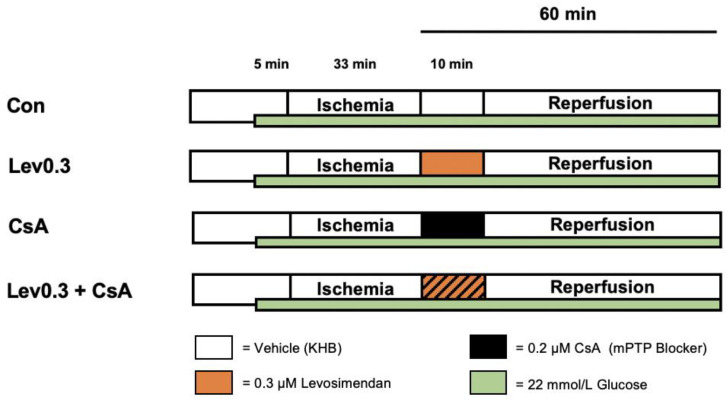
Experimental protocol. Con = Control; Lev = Levosimendan, KHB = Krebs-Henseleit-Buffer; Vehicle = Krebs-Henseleit Buffer (KHB); CsA = Cyclosporine A (mPTP inhibitor). **Green bar:** Hearts were perfused with a total of 22 mmol/L glucose concentration by combining 11 mmol/L glucose solution with KHB (containing 11 mmol/L glucose). **Control (Con):** Hearts were perfused with Krebs-Henseleit-Buffer as vehicle for 10 min. **Levosimendan 0.3 µM (Lev0.3):** Hearts were perfused with 0.3 μM Lev for 10 min. **Cyclosporine A (CsA):** Hearts were perfused with 0.2 μM CsA for 10 min. **Levosimendan 0.3 μM + Cyclosporine A (Lev0.3+CsA):** Hearts were perfused with 0.3 μM Lev and 0.2 μM CsA for 10 min.

The study consisted of two separate parts. For both parts, we employed the same basic experimental protocol (shown in Figure 3 and Figure 4). All hearts underwent 15 min of adaption period. Hereafter, acute hyperglycemia was induced in all hearts, 5 min prior to global ischemia, by applying a 11 mmol/L glucose solution consistently throughout the entire experiment. As KHB itself already contained 11 mmol/L glucose, a total of 22 mmol/L glucose was reached in all hearts. All investigations included in this study were carried out following the same protocol for induction of acute hyperglycemia. This concentration was proven to block different pharmacological conditioning strategies by us and others [22,24,35,42]. Glucose levels were frequently reevaluated throughout each experiment, by collecting coronary effluent. The current protocol was taken from our previous study, where we successfully induced acute hyperglycemia in isolated hearts [24]. Global ischemia was followed by 60 min of, including a 10 min postconditioning (PoC) period. For all experiments, global ischemia was achieved by completely stopping the perfusion to the heart via the Langendorff System. We administered the respective substance for 10 min, starting immediately at the onset of reperfusion, to achieve a postconditioning stimulus. All substances were applied at an infusion rate of 1% of coronary flow. The applied concentration of all substances in our current study were shown to be effective in several previous studies [14,59].

#### 4.2.1. Part 1: Concentration–Response Relationship of Levosimendan under Hyperglycemia

For part 1, animals were randomly assigned into 5 experimental groups (*n* = 7 per group), as shown in Figure 1. The first part was designed to investigate a possible concentration-dependent effect of Levosimendan under acute hyperglycemia. In previous studies, we demonstrated 0.3 µM as the lowest cardioprotective concentration of Levosimendan under normoglycemic conditions [14,19]. Therefore, we further increased Levosimendan concentrations to 1 µM, 3 µM, and 10 µM. Levosimendan was applied under hyperglycemic conditions as a postconditioning stimulus for 10 min after ischemia.

#### 4.2.2. Part 2: Underlying Mechanisms of Levosimendan-Induced Postconditioning under Hyperglycemia

The second part of this study was designed to elucidate the potentially underlying mechanism of blocked cardioprotection by Levosimendan, under acute hyperglycemia. Previously, we demonstrated a strong cardioprotective effect by postconditioning with 0.3 µM Levosimendan under normoglycemia, whereas higher concentrations did not further enhance the protective effect [14]. Based on these findings and results from part 1 of this study, we employed 0.3 µM Levosimendan as a postconditioning stimulus for investigations in part 2. Concentration of the mPTP inhibitor CsA was taken from the literature [59,60]. All substances were administered under hyperglycemic conditions as a postconditioning stimulus for 10 min. In part 2, hearts were randomized into 4 groups (*n* = 5 per group).

### 4.3. Statistical Analysis

#### 4.3.1. Sample Size Analysis

A sample size of *n* = 7 (part 1) and *n* = 5 (part 2) was calculated (GraphPad StatMate™, GraphPad Software, San Diego, CA, USA), detecting a 25% mean difference in infarct size (power 80%, α < 0.05 (two-tailed)).

#### 4.3.2. Statistical Approach

Both parts of the study were analyzed separately, each by performing a two-way analysis of variance (ANOVA) and a Tukey post-hoc test (GraphPad Software V7.01, San Diego, CA, USA) for hemodynamic data between groups and time-effects within each group. For part 1 and 2, infarct sizes were analyzed by a one-way ANOVA, followed by a Tukey’s post-hoc test. Data are expressed as mean ± standard deviation (SD). Changes were considered to be statistically significant if *p* < 0.05.

## 5. Conclusions

Our findings demonstrate that Levosimendan-induced postconditioning is completely abolished under hyperglycemia. Applying increased Levosimendan concentrations could not overcome blocked cardioprotective effects by hyperglycemia in this experimental setting. While inhibition of mPTP by CsA alone could not restore infarct size reduction under hyperglycemia, combining Levosimendan and CsA reversed the loss of cardioprotection under elevated glucose levels.

## Figures and Tables

**Table 1:** Weights and ischemic contracture.

		*n*	Body Weight (g)	Heart Weight Wet (g)	Heart Weight Dry (g)	Time of Max. Ischemic Contracture (min)	Level of Max. Ischemic Contracture (mmHg)
*Part 1*							
HG	Con	7	298 ± 8	1.21 ± 0.10	0.13 ± 0.01	14 ± 1	85 ± 11
	Lev0.3	7	312 ± 7	1.22 ± 0.07	0.14 ± 0.01	15 ± 2	82 ± 9
	Lev1	7	311 ± 14	1.28 ± 0.08	0.14 ± 0.01	15 ± 1	75 ± 11
	Lev3	7	313 ± 17	1.32 ± 0.07	0.14 ± 0.02	15 ± 1	82 ± 10
	Lev10	7	308 ± 13	1.22 ± 0.05	0.14 ± 0.02	16 ± 1	65 ± 8 *
*Part 2*							
HG	Con	5	316 ± 11	1.27 ± 0.04	0.14 ± 0.00	16 ± 2	75 ± 15
	Lev0.3	5	288 ± 21	1.24 ± 0.07	0.12 ± 0.01	15 ± 2	79 ± 10
	CsA	5	294 ± 24	1.15 ± 0.08	0.12 ± 0.01	15 ± 2	77 ± 7
	Lev0.3+CsA	5	307 ± 11	1.19 ± 0.04	0.12 ± 0.01	15 ± 2	68 ± 8

Data are mean ± SD, HG = Hyperglycemia; Con = Control; Lev = Levosimendan; CsA = Cyclosporine A (mPTP inhibitor); * *p* < 0.05 vs. Con.

**Table 2:** Hemodynamic variables Part 1.

		Baseline	Reperfusion		
			30	45	60
*Heart Rate (bpm)*					
HG	Con	316 ± 43	242 ± 41	219 ± 57	207 ± 69
	Lev0.3	307 ± 36	255 ± 32	228 ± 52	267 ± 43
	Lev1	288 ± 38	263 ± 109	271 ± 83	292 ± 40
	Lev3	278 ± 20	231 ± 48	208 ± 65	214 ± 45
	Lev10	282 ± 20	222 ± 64	248 ± 86	239 ± 79
*Left Ventricular Developed Pressure (mmHg)*					
HG	Con	109 ± 9	27 ± 11 *	35 ± 12 *	33 ± 6 *
	Lev0.3	107 ± 16	22 ± 14 *	25 ± 9 *	21 ± 10 *
	Lev1	117 ± 17	13 ± 10 *	20 ± 9 *	20 ± 11 *
	Lev3	114 ± 13	12 ± 12 *	20 ± 14 *	26 ± 7 *
	Lev10	122 ± 11	12 ± 10 *	19 ± 9 *	25 ± 10 *
*Coronary flow (mL/min)*					
HG	Con	12 ± 2	6 ± 1 *	6 ± 1 *	6 ± 1 *
	Lev0.3	12 ± 3	6 ± 1 *	6 ± 1 *	6 ± 1 *
	Lev1	13 ± 2	6 ± 1 *	6 ± 1 *	6 ± 1 *
	Lev3	13 ± 2	6 ± 2 *	7 ± 2 *	6 ± 2 *
	Lev10	14 ± 3	8 ± 3 *	8 ± 3 *	8 ± 3 *

Data are mean ± SD. HG = Hyperglycemia; Con = Control; Lev = Levosimendan; * *p* < 0.05 vs. Baseline.

**Table 3:** Hemodynamic variables Part 2.

		Baseline	Reperfusion		
			30	45	60
*Heart Rate (bpm)*					
HG	Con	311 ± 54	205 ± 42 *	220 ± 69	192 ± 16 *
	Lev0.3	297 ± 30	245 ± 87	259 ± 45	216 ± 52
	CsA	292 ± 23	215 ± 63	214 ± 67	229 ± 47
	Lev0.3 + CsA	306 ± 29	297 ± 43	267 ± 20	265 ± 23
*Left Ventricular Developed Pressure (mmHg)*					
HG	Con	117 ± 19	20 ± 16 *	28 ± 16 *	28 ± 11 *
	Lev0.3	106 ± 14	15 ± 13 *	20 ± 19 *	25 ± 16 *
	CsA	123 ± 26	35 ± 8 *	37 ± 13 *	35 ± 10 *
	Lev0.3 + CsA	112 ± 21	23 ± 18 *	24 ± 13 *	24 ± 12 *
*Coronary flow (mL/min)*					
HG	Con	15 ± 3	7 ± 3 *	6 ± 1 *	6 ± 2 *
	Lev0.3	13 ± 2	6 ± 1 *	6 ± 1 *	5 ± 1 *
	CsA	15 ± 2	5 ± 1 *	6 ± 1 *	6 ± 1 *
	Lev0.3 + CsA	14 ± 2	7 ± 2 *	7 ± 2 *	6 ± 1 *

Data are mean ± SD. HG = Hyperglycemia; Con = Control; Lev = Levosimendan; CsA = Cyclosporine A (mPTP inhibitor); * *p* < 0.05 vs. Baseline.

**Table 4:** Glucose levels (mg/dL).

		Baseline	Pre Ischemia	Reperfusion 15	Reperfusion 60
*Part 1*					
HG	Con	196 ± 5	370 ± 10 *	394 ± 18 *	425 ± 44 *
	Lev0.3	197 ± 6	374 ± 37 *	393 ± 11 *	425 ± 28 *
	Lev1	197 ± 2	360 ± 46 *	389 ± 13 *	409 ± 33 *
	Lev3	197 ± 4	377 ± 10 *	389 ± 32 *	393 ± 41 *
	Lev10	198 ± 3	372 ± 16 *	377 ± 13 *	400 ± 32 *
*Part 2*					
HG	Con	203 ± 3	384 ± 6 *	389 ± 12 *	418 ± 35 *
	Lev0.3	199 ± 9	373 ± 16 *	391 ± 9 *	441 ± 18 *
	CsA	204 ± 10	377 ± 9 *	390 ± 10 *	425 ± 20 *
	Lev0.3 + CsA	203 ± 6	389 ± 8 *	398 ± 5 *	448 ± 35 *

Data are mean ± SD. HG = Hyperglycemia; Con = Control; Lev = Levosimendan; CsA = Cyclosporine A (mPTP inhibitor); * *p* < 0.05 vs. Baseline.
